# Association between mental health and duty hours of postgraduate residents in Japan: a nationwide cross-sectional study

**DOI:** 10.1038/s41598-022-14952-x

**Published:** 2022-06-23

**Authors:** Kazuya Nagasaki, Yuji Nishizaki, Tomohiro Shinozaki, Taro Shimizu, Yu Yamamoto, Kiyoshi Shikino, Sho Fukui, Sho Nishiguchi, Masaru Kurihara, Koshi Kataoka, Yasuharu Tokuda, Hiroyuki Kobayashi

**Affiliations:** 1 Department of Internal Medicine, Mito Kyodo General Hospital, University of Tsukuba, 3-2-7, Miyamachi, Mito, Ibaraki 310-0015 Japan; 2 Division of Medical Education, Juntendo University School of Medicine, Tokyo, Japan; 3 Department of Information and Computer Technology, Faculty of Engineering, Tokyo University of Science, Tokyo, Japan; 4 Department of Diagnostic and Generalist Medicine, Dokkyo Medical University Hospital, Tochigi, Japan; 5 Division of General Medicine, Center for Community Medicine, Jichi Medical University, Tochigi, Japan; 6 Department of General Medicine, Chiba University Hospital, Chiba, Japan; 7 Immuno-Rheumatology Center, St. Luke’s International Hospital, Tokyo, Japan; 8 Department of General Internal Medicine, Shonan Kamakura General Hospital, Kamakura, Japan; 9 Department of Patient Safety, Nagoya University Hospital, Aichi, Japan; 10 Muribushi Okinawa for Teaching Hospitals, Okinawa, Japan

**Keywords:** Health occupations, Occupational health

## Abstract

The new duty hour (DH) limit for doctors in Japan will begin in 2024, setting the maximum DHs for postgraduate residents at approximately 80 h weekly. To set appropriate limits, understanding the association between DHs and psychological health is necessary. Thus, we assessed the relationship between residents’ psychological health and DHs. We conducted a cross-sectional study involving examinees of the General Medicine In-training Examination 2020. Mental health outcomes were measured dichotomously using the Patient Health Questionnaire-2 for depression and Mini-Z 2.0, for burnout, stress, and satisfaction. Weekly DHs were measured in seven categories at 10-h intervals. The prevalence ratios (PRs) between the DH categories were estimated for each outcome. Of the 6045 residents who provided data on DHs and psychological outcomes, 37.3% showed signs of depression, 21.6% experienced burn out, and 39.2% were highly stressed. In contrast, 62.3% were highly satisfied with their training. Proportions of burnout were higher among residents in Category 6 (≥ 90 and < 100 h; PR 1.36; 95% CI 1.11–1.66) and Category 7 (≥ 100 h; PR 1.36; 95% CI 1.10–1.68) compared with residents in Category 3 (≥ 60 and < 70 h; reference). The results partially support the weekly 80-h DH limit in terms of resident well-being.

## Introduction

Excessive working hours are a long-standing social problem in Japan, causing health problems such as depression, suicide, and *karoshi* (occupational sudden death)^1^. Doctors work particularly long hours, especially postgraduate residents and junior doctors. According to a 2018 survey by the Ministry of Health, Labor and Welfare (MHLW), 10% of all doctors work > 80 h weekly; moreover, doctors in their 20 s (mostly residents) have the longest average working hours at 76.1 h weekly^2^. The need for resident duty hour (DH) restriction led to a gradual decrease in work hours in Europe, the United States, Canada, and other regions^3^. The primary motivations for limiting DHs vary from region to region and include medical safety, human rights, and well-being.

In Japan, restrictions on residents’ DHs have been discussed from the perspective of worker well-being. The government set a uniform upper limit on working hours (up to 720 h per year of overtime) for all workers starting in 2019^4^. However, the MHLW argued for setting a different limit for doctors, to maintain the healthcare system^5^. As a result, it was decided that the upper limit for doctors should be 960 h per year of overtime work (equivalent to a 60-h work week), to be implemented in 2024. However, two exceptions were made: medical institutions that are important for maintaining community healthcare and resident doctors. Residents were allowed to work up to 1860 h of overtime per year (equivalent to an 80-h work week) for educational purposes. Members of the MHLW committee on work style reform thought that long DHs are required to maintain the quality of resident education^6^. A previous study found that resident self-study time and in-training examination scores increased until their working hours reached 60–65 h weekly^7,8^. However, this decision raised concerns about resident well-being because an 80-h limit of overtime per month is twice the standards of death due to overwork (the so-called "*karoshi* line"). However, few studies have examined DHs’ impact on health, and there is currently insufficient evidence to determine the appropriate DH limit in Japan.

Long working hours have negative effects on residents' health, and can result in increased burnout and depression^9,10^. In a cross-sectional study of 604 residents across Japan, residents experienced burnout 28.5% of the time, and residents who reported that overwork caused their stress were 2.75 times more likely to experience burnout^11^. Another study reported that doctors working ≥ 70 h weekly were 1.8 times more likely to be depressed than those working ≤ 54 h weekly^12^. A study by Ogawa et al. involving 1241 residents found that working > 80 h weekly was associated with developing depressive symptoms^13^. Occupational stress, job dissatisfaction, and short sleep are associated with burnout and poor mental health^11,12,14^. While these studies clearly demonstrate the relationship between excessively long DHs and poor mental health among Japanese residents, there are some limitations in determining appropriate DH limits. First, the numbers of residents participating in these studies were relatively small and may not be representative of all residents. Second, these studies categorized DHs into only a few categories; as a result, the point where mental health problems increase is unclear. Third, most of these studies measured only one aspect of mental health and its relationship with DHs. We expected burnout and depression to be similarly related to weekly DHs; however, stress and satisfaction may have different relationships.

This study involved a multicenter, nationwide, cross-sectional survey of postgraduate residents who took the General Medicine In-training Examination (GM-ITE), which is taken by about one-third of all residents in Japan. We evaluated the association between weekly DHs and psychological health-related outcomes (depression, burnout, job stress, and job satisfaction) to determine the appropriate DH limit.

## Methods

### Participants and data collection

We conducted a survey of postgraduate year 1 (PGY-1) and 2 (PGY-2) residents in Japan between January 18 and 31, 2020. After graduating from a 6-year medical school, students in Japan enter a 2-year postgraduate training program^15,16^. This training program is administered by the MHLW and aims to develop basic clinical skills, regardless of future specialty. Residents were assigned to a program at a community or university hospital as the core training hospital. Students can apply to the training hospital of their choice. After the 2-year program, most residents entered specialty-based training.

The survey was distributed to residents who took the GM-ITE in the 2020 academic year. The GM-ITE is an examination of general clinical knowledge and its application; used to assess individual residents and residency programs^17,18^. The Specified Nonprofit Corporation of the Japan Institute for Advancement of Medical Education Program (JAMEP) develops the examination annually with experienced doctors and peer reviewers. The exam is voluntary for each training hospital, and approximately one-third of residents take it every year. Immediately after the GM-ITE, participants completed a questionnaire to evaluate their work environment. The research consent form clearly stated that participation was voluntary and that responses would remain anonymous. All participants provided written informed consent. This study was approved by the Ethics Review Board of the JAMEP. This study was conducted in accordance with the Declaration of Helsinki.

### Measurements

The survey asked about residents’ characteristics (age, sex, PGY, and preferences of specialty), work environment factors (weekly duty hours, type of residency hospital, hospital area, experience of case-report writing or conference presentations, number of assigned inpatients, number of emergency department (ED) duties per month, and daily self-study time), and psychological health outcomes (burnout, depression, stress, and satisfaction).

The dependent variables were depression, burnout, job stress, and job satisfaction. This study was limited by the number of questions asked; thus, a brief scale was used to assess these variables. Depression was assessed by the Japanese version of the 2-item Patient Health Questionnaire (PHQ-2)^19^. The questionnaire is a simple screening tool for detecting depression and consists of two items: a loss of interest or pleasure and depressed mood over the past 2 weeks^20^. Responses were dichotomous (yes/no), and a “yes” response for either question indicated a positive depression screening. The questionnaire has a sensitivity of 0.76 and a specificity of 0.87 for identifying clinically significant depression^21^. The 10-item Mini-Z 2.0 survey was used to assess burnout, job stress, and job satisfaction. The Mini-Z evaluates doctors’ wellbeing-related outcomes and related workplace stressors^22^. Burnout, stress, and satisfaction were assessed with a single question. A single-item measure of burnout (SMB) was used to assess burnout, and respondents selected one of five options to indicate the level of burnout. The validation of the SMB was assessed in previous studies and was well correlated with emotional exhaustion and depersonalization on the Maslach Burnout Inventory, which are both core concepts of burnout^23–25^. Stress and satisfaction were rated on a 5-point Likert scale. A Japanese version of Mini-Z has been developed and validated for Japanese doctors^26^. Each question and its scoring are provided in the Appendix 1.

The independent variable of main interest was the self-reported average weekly DHs for the entire training period. It was calculated as the total of weekday work, night ED duty, and weekend work (see Appendix 1). Weekly DHs consisted of seven categories: Category 1 (< 50 h), Category 2 (≥ 50 and < 60 h), Category 3 (≥ 60 and < 70 h), Category 4 (≥ 70 and < 80 h), Category 5 (≥ 80 and < 90 h), Category 6 (≥ 90 and < 100 h), and Category 7 (≥ 100 h). The MHLW defines working hours as the time spent by a worker engaged in work at the explicit or implicit direction of the employer^6^.

### Statistical analyses

The association between resident DHs and psychological outcomes was examined in terms of PRs estimated by clustered log-linear “modified” Poisson models, in which hospital variation was accounted for as clusters in generalized estimating equations (GEEs). Considering that 60 h weekly is the basic upper limit of DHs for all doctors, Category 3 (≥ 60 and < 70 h) was used as the reference for analysis. Those who could not provide information about DHs or psychological outcomes were excluded from the analysis, because we have no auxiliary variables other than adjusted covariates in the models to impute these missing data. The models adjusted for gender, PGY, preference for specialty, hospital type, hospital region, scholarship activities, number of assigned inpatients, number of ED duties per month, and daily self-study time. As a post-hoc analysis, we explored characteristics related to resident job satisfaction among the above and other variables using the log-linear Poisson GEE model; the motivation for the analysis is indicated in the Results section. All analyses were conducted using SAS version 9.4 (Cary, NC, USA). This study followed the STROBE guidelines.

## Results

### Basic characteristics and work conditions

In total, 7669 residents from 593 teaching hospitals (total number of training hospitals: 1020 (2019) and 1017 (2020)) participated in the 2020 GM-ITE. Of these, 6816 responded to the survey for a response rate of 90%. Excluding residents with missing data, 6045 residents were used for analysis. Participants’ basic characteristics and work conditions are listed in Table 1. Of those residents, 68.1% were men, 49.2% were PGY-2, 11.3% were from university hospitals, and 32.4% were from urban hospitals. There were positive trends between DHs and men (%), community hospitals (%), number of ED duties, and number of assigned inpatients.

**Table 1:** Basic characteristics and work conditions of postgraduate residents classified by duty hours.

	Total	C1: < 50 h	C2: ≥ 50 and < 60 h	C3: ≥ 60 and < 70 h	C4: ≥ 70 and < 80 h	C5: ≥ 80 and < 90 h	C6: ≥ 90 and < 100 h	C7: ≥ 100 h
	N = 6045	N = 861	N = 1687	N = 1580	N = 709	N = 741	N = 258	N = 209
***Gender (%)***								
Men	68.1	64.0	68.5	67.7	70.7	68.3	57.1	76.1
Women	31.9	36.0	31.5	32.3	29.3	31.7	42.9	23.9
***Postgraduate year (%)***								
PGY-1	50.8	51.3	51.4	50.4	50.2	51.0	51.9	48.3
PGY-2	49.2	48.7	48.6	49.7	49.8	49.0	48.1	51.7
***Hospital type (%)***								
University	11.3	17.0	11.7	10.5	10.7	8.6	7.0	6.2
University-affiliated	5.0	5.6	4.7	5.2	4.1	5.5	5.8	2.9
Community	83.8	77.5	83.6	84.3	85.2	85.8	87.2	90.9
***Hospital area (%)***								
Urban	32.4	32.5	30.4	32.5	36.5	33.6	32.2	30.1
Non-urban	67.6	67.5	69.6	67.5	63.5	66.4	67.8	69.9
***Career (%)***								
Internal medicine	35.8	33.4	36.4	36.1	36.0	35.6	32.9	42.1
Surgery	20.8	20.2	18.3	21.3	21.3	25.5	19.8	21.1
General medicine	2.4	2.3	2.9	2.3	2.1	1.9	2.7	2.9
Emergency medicine	3.4	3.3	2.3	3.5	3.7	4.7	5.0	5.7
Others	37.5	40.8	40.1	36.8	36.7	32.3	39.1	28.2
***Scholarship activity (%)***								
Case report	21.2	21.7	18.6	21.1	22.1	23.8	22.9	27.3
Presentation at conferences	37.1	33.2	36.0	37.7	37.7	40.4	41.1	37.8
***ED duties per month (%)***								
0–1	3.7	9.5	4.6	2.7	1.8	0.9	0.8	0.5
1–2	15.3	25.9	17.7	12.2	12.6	12.4	7.8	5.3
3–5	70.2	59.5	71.9	75.6	70.7	69.5	68.6	63.6
6 or more	10.2	4.2	5.5	9.1	14.4	16.7	22.5	29.2
Unknown	0.5	0.8	0.4	0.4	0.6	0.4	0.0	1.4
***Assigned inpatients (%)***								
0–4	23.5	33.8	28.0	22.8	18.2	14.3	15.1	10.0
5–9	59.9	55.5	61.5	59.6	63.0	63.0	55.4	49.3
0–14	11.3	7.8	7.1	12.4	14.0	13.9	20.5	21.5
15 or more	2.9	0.7	0.9	2.3	3.1	5.5	6.6	16.7
Unknown	2.5	2.2	2.5	2.8	1.6	3.2	2.3	2.4
***Self-study time per day (%)***								
None	3.5	3.8	3.3	3.8	2.5	4.0	2.7	3.8
0–30 min	34.1	41.9	36.8	32.8	31.9	26.3	30.6	28.2
31–60 min	41.5	39.8	41.4	42.2	42.0	42.4	40.7	39.2
61–90 min	16.7	12.1	14.8	17.2	17.9	22.8	19.4	17.7
91 min or more	4.3	2.3	3.7	3.9	5.6	4.3	6.6	11.0

The average weekly duty hours are based on the sum of weekday work duties, night ED duties, and weekend work duties. Abbreviations: C1–C7, Category 1 to Category 7; h, hours; PGY, postgraduate year; ED, emergency department.

### Psychological health-related outcomes

**Figure 1:**
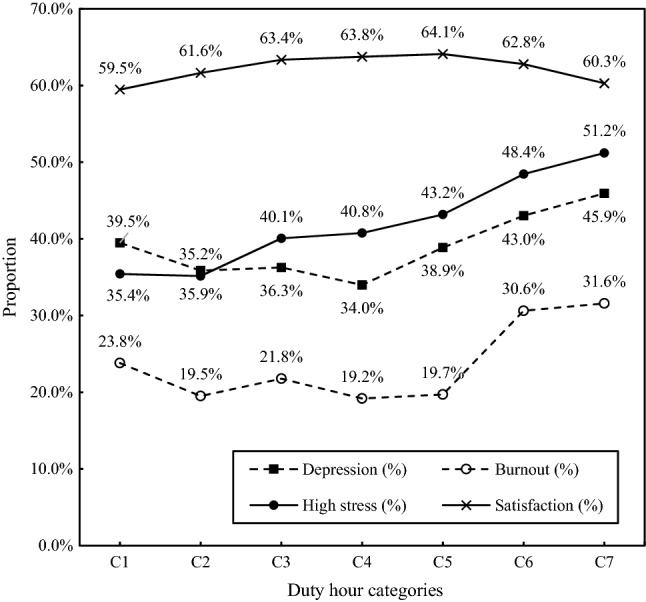
The association between duty hours and psychological health-related outcomes. *Note* Figure shows that depression, burnout, and high stress among residents appeared to increase with DHs; however, this was not associated with satisfaction. The categories of resident duty hours weekly were: Category 1 (< 50), Category 2 (≥ 50 and < 60), Category 3 (≥ 60 and < 70), Category 4 (≥ 70 and < 80), Category 5 (≥ 80 and < 90), Category 6 (≥ 90 and < 100), and Category 7 (≥ 100). Abbreviations: C1–C8 = Category 1 to Category 8.

Among participants, 37.3% experienced depression, 21.6% experienced burnout, and 39.2% had high job stress. In contrast, 62.3% were satisfied with their jobs. Residents who worked more hours were more likely to experience depression, burnout, and high levels of stress; however, this was not associated with satisfaction (Fig. 1). Among the residents working > 100 h weekly, 45.9% were depressed, 31.6% experienced burn out, and 51.2% were highly stressed.

Table 2 shows the PRs of mental health-related outcomes among DH categories. Compared to residents in Category 3 (≥ 60 and 70 h; reference), residents in Category 7 (≥ 100 h; PR 1.21 from the multivariable-adjusted model; 95% CI 1.04–1.41) were more likely to experience depression. The same trend was observed for burnout, and there was more depression among residents in Categories 6 (≥ 90 and < 100 h; PR 1.36; 95% CI 1.11–1.66) and 7 (PR 1.36; 95% CI 1.10–1.68) than Category 3. Excessive job stress was common among residents in Categories 6 (PR 1.20; 95% CI 1.04–1.38) and 7 (PR 1.26; 95% CI 1.07–1.48), but less common among residents in Categories 1 (< 50 h; PR 0.86; 95%CI 0.77–0.96) and 2 (≥ 50 and < 60 h; PR 0.87; 95% CI 0.80–0.95) compared to the reference. There was no difference in job satisfaction among the DH categories.

**Table 2:** Association between resident duty hours and mental health outcomes (n = 6045).

Outcome	DH category	*N*	Outcome prevalence (%)	Univariable model				Multivariable model^†^			
				PR	95% CI		*P*	PR	95% CI		*P*
Depression	C1: < 50 h	861	39.5	1.09	0.98	1.22	0.128	1.07	0.96	1.20	0.213
	C2: ≥ 50 and < 60 h	1687	35.9	0.99	0.90	1.08	0.807	0.99	0.90	1.08	0.829
	C3: ≥ 60 and < 70 h	1580	36.3	1	Reference	1	Reference				
	C4: ≥ 70 and < 80 h	709	34.0	0.94	0.83	1.06	0.297	0.94	0.83	1.06	0.290
	C5: ≥ 80 and < 90 h	741	38.9	1.07	0.96	1.20	0.222	1.07	0.96	1.20	0.222
	C6: ≥ 90 and < 100 h	258	43.0	1.19	1.02	1.38	0.030	1.15	0.99	1.34	0.061
	C7: ≥ 100 h	209	45.9	1.27	1.08	1.49	0.004	1.21	1.04	1.41	0.016
Burnout	C1: < 50 h	861	23.8	1.09	0.94	1.27	0.231	1.05	0.90	1.22	0.542
	C2: ≥ 50 and < 60 h	1687	19.5	0.90	0.78	1.03	0.119	0.88	0.77	1.02	0.082
	C3: ≥ 60 and < 70 h	1580	21.8	1	Reference	1	Reference				
	C4: ≥ 70 and < 80 h	709	19.2	0.88	0.74	1.04	0.145	0.87	0.73	1.03	0.107
	C5: ≥ 80 and < 90 h	741	19.7	0.90	0.76	1.08	0.259	0.91	0.77	1.09	0.303
	C6: ≥ 90 and < 100 h	258	30.6	1.41	1.15	1.72	0.001	1.36	1.11	1.66	0.003
	C7: ≥ 100 h	209	31.6	1.45	1.17	1.80	0.001	1.36	1.10	1.68	0.005
High stress	C1: < 50 h	861	35.4	0.88	0.79	0.98	0.025	0.86	0.77	0.96	0.007
	C2: ≥ 50 and < 60 h	1687	35.2	0.88	0.80	0.96	0.004	0.87	0.80	0.95	0.002
	C3: ≥ 60 and < 70 h	1580	40.1	1	Reference	1	Reference				
	C4: ≥ 70 and < 80 h	709	40.8	1.02	0.92	1.13	0.743	1.02	0.92	1.14	0.643
	C5: ≥ 80 and < 90 h	741	43.2	1.08	0.98	1.19	0.134	1.09	0.99	1.20	0.083
	C6: ≥ 90 and < 100 h	258	48.4	1.21	1.04	1.40	0.012	1.20	1.04	1.38	0.013
	C7: ≥ 100 h	209	51.2	1.28	1.09	1.50	0.003	1.26	1.07	1.48	0.005
Satisfaction	C1: < 50 h	861	59.5	0.94	0.88	1.00	0.044	0.97	0.92	1.04	0.407
	C2: ≥ 50 and < 60 h	1687	61.6	0.97	0.92	1.03	0.327	0.99	0.94	1.04	0.643
	C3: ≥ 60 and < 70 h	1580	63.4	1	Reference	1	Reference				
	C4: ≥ 70 and < 80 h	709	63.8	1.01	0.94	1.08	0.853	1.00	0.93	1.06	0.880
	C5: ≥ 80 and < 90 h	741	64.1	1.01	0.95	1.08	0.725	0.99	0.93	1.06	0.768
	C6: ≥ 90 and < 100 h	258	62.8	0.99	0.90	1.09	0.858	0.97	0.88	1.07	0.540
	C7: ≥ 100 h	209	60.3	0.95	0.85	1.07	0.411	0.92	0.82	1.04	0.176

There was a higher proportion of burnout and depression among residents with DHs > 90 h weekly. High stress was positively correlated with DH; however, satisfaction was not. ^†^Adjusted for gender, postgraduate year, hospital type, hospital region, specialty preference, scholarship activities (case report or conference presentation), number of assigned inpatients, number of ED duties per month, and self-study time per day.

DH, duty hour; PR, prevalence ratio; CI, confidence interval; C1–C8, Categories 1 to 8; h, hours.

### Resident job satisfaction and associated factors

No association was observed between job satisfaction and DHs. In our post-hoc analysis, PGY-2 increased satisfaction, but hospital type and sex had no effect on satisfaction (Table 3). Residents who chose internal medicine, surgery, general medicine, and emergency medicine as their career aspirations were more satisfied than those who chose “others.” More-satisfied residents were more likely to spend more time on self-study, and those who received support for conference presentations were more satisfied. The number of ED night duties was not related to satisfaction, nor were DHs. Low satisfaction was observed among residents assigned < 0**–**4 inpatients.

**Table 3:** Estimates of the ratios of proportions satisfied among residents' and their hospitals' characteristics.

	Prevalence ratio	95% CI	P value
***Men (vs. Women)***	0.97	0.93–1.02	0.248
***PGY-2 (vs. PGY-1)***	1.07	1.02–1.11	0.005
***Hospital type***			
Community	Reference		
University	0.93	0.85–1.01	0.085
University-affiliated	0.91	0.79–1.05	0.187
***Urban***	0.98	0.94–1.03	0.527
***Career preference***			
Internal medicine	Reference		
Surgery	1.01	0.95–1.06	0.815
General medicine	1.01	0.89–1.15	0.842
Emergency medicine	1.01	0.39–1.13	0.790
Others	0.90	0.86–0.95	0.000
***Case report (yes vs. no)***	0.97	0.92–1.01	0.165
***Presentation at conferences (yes vs. no)***	0.95	0.90–0.99	0.017
***Self-study time***			
0–30 min	Reference		
31–60 min	1.09	1.04–1.15	0.000
61–90 min	1.19	1.13–1.25	0.000
91 min or more	1.20	1.10–1.31	0.000
None	0.72	0.61–0.86	0.000
***Night ED duty***			
0	Reference		
1–2	0.91	0.78–1.06	0.215
3–5	0.97	0.84–1.11	0.638
6 or more	0.96	0.82–1.11	0.567
Unknown	0.72	0.45–1.13	0.152
***Assigned inpatients***			
0–4	Reference		
5–9	1.08	1.02–1.14	0.004
10–14	1.10	1.02–1.19	0.014
15 or more	0.99	0.86–1.13	0.836
Unknown	0.87	0.74–1.03	0.108

PGY, postgraduate year; ED, emergency department.

## Discussion

This is the largest study in Japan to examine the relationship between DHs and resident mental health. In this study, weekly DHs were assessed in 10-h intervals, and findings showed that working > 90 h weekly increased burnout and depression among residents. We also found that long DHs were associated with excessive job stress. However, job satisfaction was unrelated to DHs, and the major factors for low satisfaction were specialty orientation and less self-study time.

The present study showed that depression increase when working > 90 h weekly. Ogawa et al. and Tomioka et al. reported that depression increased in residents who worked > 80 h and > 70 h weekly, respectively^12,13^. The results of the present study generally support those findings. It is a new finding that burnout, like depression, also increases with DHs > 90 h weekly; however, although burnout and depression are considered distinct concepts, they are conceptually similar in some ways, and it is possible that we are observing the same condition^27^. In our study, stress was shown to be positively correlated with DHs. In a nationwide survey of Japanese residents, 26.0% of burned-out residents reported excessive work hours as stressful^11^. Residents who are at high risk for burnout may need to avoid working long hours. In addition, the difference between this study and previous studies is that this study was conducted during the COVID-19 pandemic. During this pandemic, clinicians, including residents, were subjected to considerable psychological stress, which led to depression and burnout^28^. In a study of Japanese healthcare workers, younger age and fewer years of experience were associated with increased burnout, which may have influenced the results of this study^29^. On the other hand, some studies have reported that the pandemic did not necessarily increase working hours but led to a decrease in working hours^30^. Although not reported as a study, many hospitals in Japan removed residents from the COVID-19 front line as part of infection-control measures. Therefore, the pandemic’s effect on the results of this study is unclear, and further research is needed.

As there was no significant relationship between job satisfaction and DHs in this study, we conducted a post-hoc analysis to determine which factors were related to satisfaction. Although there are few studies on resident satisfaction in Japan, one study found that men, scholarly activity, good compensation, and favorable curriculum were associated with higher satisfaction^31^. In our analysis, the most notable factors for low satisfaction were career preference for "others" rather than internal medicine, surgery, general medicine, or emergency medicine. The "others" are fields that require more specific specialty skills than those listed above, meaning that residents who chose these fields are specialty-oriented. The low satisfaction level among specialty-oriented residents is thought to be because the 2-year postgraduate training is generalist-oriented, with an emphasis on acquiring general clinical skills. However, factors related to workload, such as DHs and number of night ED duties, were not related to satisfaction level. Since generalist-oriented students are willing to spend a long time learning during their training period, it is thought that their satisfaction level does not decrease. Since there is a strong relationship between satisfaction and the length of self-study time in this study, we need to determine how to increase the satisfaction of residents with low satisfaction. However, since this is a cross-sectional study, the causal relationship between resident satisfaction and each factor is unknown.

How do these results impact the setting of maximum DHs for residents in Japan? Starting in 2024, overtime work for all doctors will be limited to 960 h per year (equivalent to a 60-h work week), and overtime work for doctors in training will be limited to a maximum of 1860 h per year (equivalent to an 80-h work week)^5,6^. Combining the current results with the results of previous studies on DHs^7,8,12,13^, we have illustrated the differences in mental health and other outcomes in Table 4 by DHs into three groups: < 60 h weekly, 60–80 h weekly, and > 80 h weekly. The conclusion that can be drawn from this table is that working > 80 h weekly negatively impacts mental health, whereas working < 60 h weekly negatively impacts education. Therefore, the results of these studies provide a reasonable explanation for the MHLW policy, which allows residents to work up to 80 h weekly. Additionally, 80 h of work per week should be the upper limit of working hours in countries other than Japan as well for protecting the health of resident physicians. In countries without an upper limit on DHs or an upper limit that exceeds 80 h per week, policy makers might need to conduct research on resident health and reconsider settings for upper limits on DHs.

**Table 4:** The summary of evidence related to duty hours and postgraduate residents in Japan.

	Weekly duty hours		
	< 60 h	60–80 h	> 80 h
**Mental health (our study)**			
Burnout^11^	→	→	↑
Depression^12,13^	→	→	↑
Stress	↓	→	↑
Satisfaction	→	→	→
**Education**			
In-training exam score^8^	↓	→	→
Self-study time^7^	↓	→	→
Patient safety	?	?	?

This table shows the factors associated with duty hours of postgraduate residents in Japan. Using 60–80 h weekly (equivalent to 80–120 h of overtime per month) as a reference point, the table shows how these factors change when working less than 60 h weekly or more than 80 h weekly. The references corresponding to each item are presented in the table.

Several additional issues, however, must be considered to set the upper limit of DH. First, it is necessary to consider the issue in terms of medical safety^3,32,33^. Doctors and residents’ long working hours threaten patient safety worldwide, and an investigation of the extent of their impact in Japan is strongly encouraged. Next, there is a large range of overtime hours, from 960 to 1860 h per year (equivalent to 60–80 h weekly); further study is needed to determine the acceptable level of working hours. Finally, there is a question of whether this limit is appropriate for all residents. The analyses of satisfaction showed that residents were not necessarily homogenous and had different needs. Reducing the upper limit of 1860 h per year may be an option to prevent specialty-oriented residents from receiving long hours of unwanted training.

This study has several limitations. First, it assessed mental health-related outcomes using simple scales that did not confirm the diagnosis of burnout or depression. These scales were used to reduce the burden on residents, as this study was conducted after the examination. In this study, burnout was assessed with the SMB; however, one study demonstrated that self-diagnosis with the SMB reported less burnout than the Maslach Burnout Inventory, which is the gold standard for diagnosis of burnout^24^. Therefore, it is possible that we may have underestimated actual burnout. In addition, as noted above, the SMB does not correlate with reduced personal accomplishment, which is one factor in burnout. Depression was assessed using the PHQ-2, which is only a screening tool. Second, the DHs in this study were self-reported, and may not have been accurate. In addition, the study did not consider the differences between DHs in each rotation, but rather examined the average DHs for the entire training period. Therefore, even if a resident's DHs are of short durations overall, only some rotations may have outstandingly long DHs, which may cause mental health problems. Third, we did not have data on participants' underlying mental health problems or personalities. This is important because mental health problems during medical school have been reported to be a predictor of the onset of depression and burnout during training^34^. Fourth, only one-third of all residents participated in the GM-ITE, which may have led to selection bias. Participation in the GM-ITE is voluntary for training hospitals that emphasize education. Residents with mood or anxiety disorders may tend not to select such hospitals because they value the work environment more than education. In addition, approximately 10% of examinees who took the GM-ITE were excluded from the analysis due to missing data, which may also be related to selection bias. However, the basic characteristics of the residents excluded from the study were similar to those of the study participants, limiting the impact of this bias (Appendix 2). Fifth, it is not possible to prove a causal relationship between DH and psychological outcomes due to the nature of the cross-sectional study. However, this study was designed to assess the impact of DHs on mental health by considering the temporal relationship between "past DH" and "current mental health status.” Moreover, since both items were to be responded to at the same time, there may be response bias in both responses depending on actual mental health status. Follow-up studies monitoring DHs and mental health status in the short to medium term are desirable.

In conclusion, these results suggest that depression, burnout, and job stress may increase in postgraduate residents with DHs ≥ 90 h weekly. This study partially supports the 80-h work week limit in terms of resident well-being, but this limit should also be considered from the perspectives of resident education, medical safety, and workers' rights.

## Supplementary Information

Supplementary Information.

## Data Availability

Due to the nature of this research, participants of this study did not agree for their data to be shared publicly, so supporting data is not available.

## References

[1] Hiyama T, Yoshihara M (2008). New occupational threats to Japanese physicians: Karoshi (death due to overwork) and karojisatsu (suicide due to overwork). Occup. Environ. Med..

[2] Ministry of Health, Labour and Welfare. Survey on physicians’ working conditions and working style intentions. https://www.mhlw.go.jp/file/05-Shingikai-10801000-Iseikyoku-Soumuka/0000161146.pdf (**in Japanese**).

[3] Imrie KR, Frank JR, Parshuram CS (2014). Resident duty hours: Past, present, and future. BMC Med. Educ..

[4] Ministry of Health, Labour and Welfare. Act on the arrangement of related acts to promote work style reform. https://www.mhlw.go.jp/content/000332869.pdf (**in Japanese**).

[5] Tsutsumi, A. Workstyle reform for Japanese doctors. *EOH-P* (2020). 10.1539/eohp.2020-0008-OP.

[6] Ministry of Health, Labour and Welfare. Addressing the reform of the working style of physicians in the clinical training system. https://www.mhlw.go.jp/content/10803000/000590866.pdf (**in Japanese**).

[7] Nagasaki K, Nishizaki Y, Shinozaki T, Kobayashi H, Tokuda Y (2021). Association between resident duty hours and self-study time among postgraduate medical residents in Japan. JAMA Netw. Open.

[8] Nagasaki K (2021). Impact of the resident duty hours on in-training examination score: A nationwide study in Japan. Med. Teach..

[9] Lefebvre DC (2012). Perspective: Resident physician wellness: A new hope. Acad. Med..

[10] Kijima S, Tomihara K, Tagawa M (2020). Effect of stress coping ability and working hours on burnout among residents. BMC Med. Educ..

[11] Matsuo T, Takahashi O, Kitaoka K, Arioka H, Kobayashi D (2021). (2021) Resident burnout and work environment. Intern. Med..

[12] Tomioka K, Morita N, Saeki K, Okamoto N, Kurumatani N (2011). Working hours, occupational stress and depression among physicians. Occup. Med. (Lond.).

[13] Ogawa R (2018). The relationship between long working hours and depression among first-year residents in Japan. BMC Med. Educ..

[14] Tokuda Y (2009). The interrelationships between working conditions, job satisfaction, burnout and mental health among hospital physicians in Japan: A path analysis. Ind. Health.

[15] Kozu T (2006). Medical education in Japan. Acad. Med..

[16] Teo A (2007). The current state of medical education in Japan: A system under reform. Med. Educ..

[17] Nishizaki Y (2020). Impact of general medicine rotation training on the in-training examination scores of 11, 244 Japanese resident physicians: A nationwide multi-center cross-sectional study. BMC Med. Educ..

[18] Nagasaki K (2021). Validation of the general medicine in-training examination using the professional and linguistic assessments board examination among postgraduate residents in Japan. Int. J. Gen. Med..

[19] Muramatsu K (2007). The patient health questionnaire, Japanese version: Validity according to the mini-international neuropsychiatric interview–plus. Psychol. Rep..

[20] Kroenke K, Spitzer RL, Williams JBW (2003). The patient health Questionnaire-2: Validity of a two-item depression screener. Med. Care.

[21] Manea L (2016). Identifying depression with the PHQ-2: A diagnostic meta-analysis. J. Affect. Disord..

[22] Linzer M (2016). Worklife and wellness in academic general internal medicine: Results from a national survey. J. Gen. Intern. Med..

[23] Rohland BM, Kruse GR, Rohrer JE (2004). Validation of a single-item measure of burnout against the Maslach Burnout Inventory among physicians. Stress Health.

[24] Knox M, Willard-Grace R, Huang B, Grumbach K (2018). Maslach Burnout Inventory and a SELF-defined, single-item burnout measure produce different clinician and staff burnout estimates. J. Gen. Intern. Med..

[25] Nagasaki K, Seo E, Maeno T, Kobayashi H (2022). Diagnostic accuracy of the Single-item Measure of Burnout (Japanese version) for identifying medical resident burnout. J. Gen. Fam. Med..

[26] Nagasaki K (2021). (2021) Translation, cultural adaptation, and validation of the mini-Z 2.0 survey among Japanese physicians and residents. Intern. Med..

[27] Bianchi R, Schonfeld IS, Laurent E (2015). Burnout-depression overlap: A review. Clin. Psychol. Rev..

[28] Shapiro J, McDonald TB (2020). Supporting clinicians during Covid-19 and beyond—Learning from past failures and envisioning new strategies. N. Engl. J. Med..

[29] Matsuo T (2021). Health care worker burnout after the first wave of the coronavirus disease 2019 (COVID-19) pandemic in Japan. J. Occup. Health.

[30] Hu X, Dill MJ (2021). Changes in physician work hours and patterns during the COVID-19 pandemic. JAMA Netw. Open.

[31] Takahashi O (2009). Residents’ experience of scholarly activities is associated with higher satisfaction with residency training. J. Gen. Intern. Med..

[32] Fletcher KE, Reed DA, Arora VM (2011). Patient safety, resident education and resident well-being following implementation of the 2003 ACGME duty hour rules. J. Gen. Intern. Med..

[33] Bolster L, Rourke L (2015). The effect of restricting residents’ duty hours on patient safety, resident well-being, and resident education: An updated systematic review. J. Grad. Med. Educ..

[34] Sen S (2010). A prospective cohort study investigating factors associated with depression during medical internship. Arch. Gen. Psychiatry.

