# Supplementary material for: Association between mental health and duty hours of postgraduate residents in Japan: a nationwide cross-sectional study
Source: Sci Rep. 2022 Jun 23;12:10626. doi: 10.1038/s41598-022-14952-x (PMC9218701; doi:10.1038/s41598-022-14952-x)
Supplement: Supplementary file 1 — Supplementary Information. [file 41598_2022_14952_MOESM1_ESM.pdf]

Appendices for "Association between mental health and duty hours of postgraduate residents in Japan: A nationwide cross-sectional study"

These appendices consist of the following two sections.

Appendix 1. Questions about the psychological health-related outcomes in the questionnaire accompanied by the General Medicine In-training Examination (GM-ITE)

Appendix 2. Basic characteristics and work conditions of postgraduate residents who are excluded from the analysis

Appendix 1. Questions about the psychological health-related outcomes in the questionnaire accompanied by the General Medicine In-training Examination (GM-ITE)

In this section, we present questions about the study variables. First, we present the 2-item Patient Health Questionnaire (PHQ-2) to measure depression. Second, we describe 1-item questions pertaining to burnout, stress, and satisfaction and their scorings, which are included in the Mini-Z. Last, we show a question about the calculation of average duty hours.

Depression

Have you been feeling down or depressed often during the past month?

(1) Yes

(2) No

Have you often felt uninterested or not very pleased with doing things during the past month?

(3) Yes

(4) No

Scoring

If either question is yes, the respondent is considered positive for depression screening.

Burnout, Stress, and Satisfaction

Burnout

Please select the one that best describes your situation based on your own definition of "burnout."

(1) You feel totally burned out. You are at a point when you may need some help.

(2) You always have symptom(s) of burnout. You are often worried about stress from work.

(3) You are beginning to experience burnout and have at least one symptom of burnout (e.g., emotional exhaustion).

(4) You feel under stress. You are not always full of energy, but have never felt burned out.

(5) You enjoy working. You have never felt burned out.

Stress

You feel/have felt under strong stress from work.

(1) Strongly agree

(2) Agree

(3) Neither agree nor disagree

(4) Disagree

(5) Strongly disagree

Satisfaction

Generally, you are satisfied with your current work.

(1) Strongly disagree

(2) Disagree

(3) Neither agree nor disagree

(4) Agree

(5) Strongly agree

Scoring

Burnout: 1-3 = burnout

Stress: 1-2 = high stress

Satisfaction: 4-5 = satisfied

Average duty hours per week (including standby hours in emergency duty)

(1) <50 hours

(2) 50–60 hours

(3) 60–70 hours

(4) 70–80 hours

(5) 80-90 hours

(6) 90–100 hours

(7) >100 hours

Note: Example of calculation

Weekday duty hours (Monday – Friday, 8:30–19:00, excluding 1 hour break): 47.5 hours

Weekday night emergency duty (1 time per week): 13.5 hours

Weekend duty hours + Night emergency duty (1 time per week): 24 hours

= 85 hours

Appendix 2. Basic characteristics and work conditions of postgraduate residents who are excluded from the analysis

	Excluded data
	N=771
Demographics:	
Men (%)	69.8
PGY-2 (%)	48.9
Hospital types (%):	
University	8.3
University branch	6.6
Community	85.1
Hospital area (%):	
Urban	37.4

Abbreviations: PGY = postgraduate year.
